# The Saliency of Snake Scales and Leopard Rosettes to Infants: Its Relevance to Graphical Patterns Portrayed in Prehistoric Art

**DOI:** 10.3389/fpsyg.2021.763436

**Published:** 2021-11-22

**Authors:** Richard G. Coss, Eric P. Charles

**Affiliations:** Psychology Department, University of California, Davis, Davis, CA, United States

**Keywords:** crosshatch patterns, human infants, nonhuman primates, leopard perception, snake perception, prehistoric engraving

## Abstract

Geometrically arranged spots and crosshatched incised lines are frequently portrayed in prehistoric cave and mobiliary art. Two experiments examined the saliency of snake scales and leopard rosettes to infants that are perceptually analogous to these patterns. Experiment 1 examined the investigative behavior of 23 infants at three daycare facilities. Four plastic jars (15×14.5cm) with snake scales, leopard rosettes, geometric plaid, and plain patterns printed on yellowish-orange paper inside were placed individually on the floor on separate days during playtime. Fourteen 7–15-month-old infants approached each jar hesitantly and poked it before handling it for five times, the criterion selected for statistical analyses of poking frequency. The jars with snake scales and leopard rosettes yielded reliably higher poking frequencies than the geometric plaid and plain jars. The second experiment examined the gaze and grasping behavior of 15 infants (spanning 5months of age) seated on the laps of their mothers in front of a table. For paired comparisons, the experimenter pushed two of four upright plastic cylinders (13.5×5.5cm) with virtually the same colored patterns simultaneously toward each infant for 6s. Video recordings indicated that infants gazed significantly longer at the cylinders with snake scales and leopard rosettes than the geometric plaid and plain cylinders prior to grasping them. Logistic regression of gaze duration predicting cylinder choice for grasping indicated that seven of 24 paired comparisons were not significant, all of which involved choices of cylinders with snake scales and leopard rosettes that diverted attention before reaching. Evidence that these biological patterns are salient to infants during an early period of brain development might characterize the integration of subcortical and neocortical visual processes known to be involved in snake recognition. In older individuals, memorable encounters with snakes and leopards coupled with the saliency of snake scales and leopard rosettes possibly biased artistic renditions of similar patterns during prehistoric times.

## Introduction

To explore how the visual system might foster graphical expression, we investigate infants’ responses to the camouflaging patterns of two dangerous animals: snakes and leopards. Background-matching camouflage can be an effective way predators avoid detection by prey. However, such camouflage has fostered an evolutionary arms race, in which natural selection for predator detection has led some prey to capitalize on the historical reliability of camouflaging features by converting them into effective predator recognition cues (Coss et al., 2005).

The process of how visual imagery is translated into visuomotor guidance of the hand during the production of artworks has received little study, but likely involves an interaction of innate perceptual processes and learning. In this paper, we will argue that intense experiences with snakes and large-bodied felids enhancing memory might have influenced the generation of graphical patterns that characterize these visual cues. With learning boosting levels of saliency, the innate properties of pattern recognition would continue to act as perceptual scaffolding for this endeavor (*cf.* Coss, 1968, 2003, 2020). As such, an evaluation of how infants respond to snake scales and leopard rosettes might shed light on the development of this relationship.

### Paleolithic Graphic Patterns

**Figure 1:**
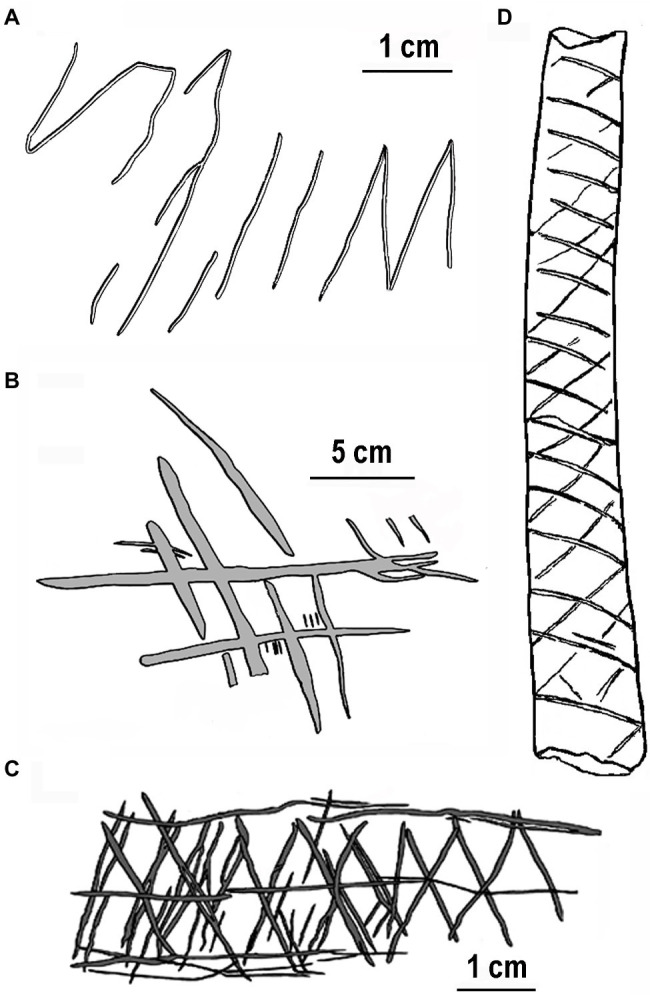
Earliest engravings by hominins redrawn for clarity: **(A)** freshwater shell, Asian *Homo erectus* ~500ka, **(B)** Gorham’s Cave, *Homo neanderthalensis* ~39ka, **(C)** Blombos Cave, *Homo sapien* ~70ka, and **(D)** Vogelherd Cave, *Homo sapien* ~34ka. Note the relatively even spacing between lines.

We begin by describing four of the early engravings that characterize the special property of repetitively engraved lines in a progression of hominin evolution. The oldest known engraving discovered in Trinil, Java, and Indonesia (Joordens et al., 2015) was produced by an early hominin, *Homo erectus*, on a freshwater shell tool between 540 and 430ka (1,000years ago). Possibly engraved using a shark’s tooth requiring considerable force, this design includes a series somewhat regularly spaced parallel lines that contain an “M” shape, all of which were made at the same time (Figure 1A). Prior to shell fossilization, this engraving would have been white contrasted by a dark-brown background. A more organized engraving showing visuomotor advancement was made a presumed Neanderthal (*Homo neanderthalensis*) on an elevated platform in Gorham’s cave, Gibraltar, older than 39ka (Rodríguez-Vidal et al., 2014). This engraving consists of deeply engraved oblique lines yielding a crisscross or crosshatch design depending on the viewing angle (Figure 1B). Ten incised lines are virtually parallel, six of which are almost equally spaced, a property requiring careful deliberation of line spacing. Related to the parallel lines of this image, equal spacing of engraved orthogonally intersecting lines (chevron pattern) is more apparent on a giant deer phalanx found at a cave entrance in northern Germany and dated at approximately 51ka (Leder et al., 2021). The Middle Paleolithic context in which this bone was found is clearly linked to Neanderthals. Although somewhat more simplified than the engraving in Gorham’s cave, this design exhibits more geometric regularity requiring considerable precision in line spacing equivalent to the many Upper Paleolithic works of modern humans (*Homo sapiens*) in Europe (e.g., Marshack, 1972; Conard, 2011).

Earlier engravings with more complex designs by modern humans (*H. sapiens*) occur in the Middle Stone Age of Africa. For example, several engraved pieces of red ochre with crosshatch designs were found in Blombos Cave, South Africa, dated at over 70ka (Henshilwood et al., 2002). One example (Figure 1C), with serially crosshatched lines with a centered horizontal line and bordered by horizontal lines, superficially resembles snake-scale tessellation (see discussion in Coss, 2003, p. 109). The repetitive properties of ventral snake scales might have influenced the ladder-like designs engraved on ostrich shells ~60ka found at Diepkloof rock shelter, South Africa (Texier et al., 2010). A more convincing snake-like engraved pattern with repetitive crisscrosses (Figure 1D) appears on a decorated bone from Vogelherd Cave by Upper Paleolithic Aurignacian hunter-gatherers in southwestern Germany (Conard and Bolus, 2003).

While these images are selectively emphasized due to their repetitive parallel lines and crisscrossing patterns, other designs include organized spots (dots) that are analogous to flecks, spots, and rosettes of felid predators that have camouflaging properties in dappled light (Coss et al., 2005). For example, clusters of dots can resemble the spots on animal pelage as evident in the realistic depictions of a leopard in Chauvet Cave, Ardèche, France (Spassov and Stoytchev, 2005) and a horse in Pech-Merle Cave, France (Pruvost et al., 2011). Dots are evident in Upper Paleolithic cave art and engraved mobiliary art (Marshack, 1972), although a prominent example of a multi-columnar cluster of red dots in La Pasiega Cave, Spain, originally attributed to Neanderthals (Hoffmann et al., 2018), is now heavily contested (*cf.* Hoffmann et al., 2020; White et al., 2020). Dots in organized arrays might have symbolic properties (Lewis-Williams and Clottes, 1998) or may be the precursors to more advanced figurative art (Hodgson and Pettitt, 2018) and even as a form of writing (von Petzinger, 2017). Wade (2006a) argues that there are so many different patterns of dots in cave art that, although some configurations might characterize prehistoric star maps, most interpretations of geometric dot arrangements are highly speculative. Wade (2006b) further suggests that, as possible abstract symbols, dots in cave art are frequently painted in red, enhancing their visibility in dim light to dark-adapted eyes. We will now argue that prehistoric crosshatch and dotted designs achieved their saliency as graphical representations because they characterized the perceptual features of dangerous animals essential to detect for survival purposes.

### Snake-Scale Saliency

Repetitive crosshatching, zigzags, and dot patterns are not frequently seen in vegetation (e.g., the Brazilian rattlesnake plant *Geoppertia insignis*), but they do occur as camouflaging patterns on snake scales and on some felids (Coss, 2003; Isbell, 2006). There is an emerging body of literature describing how different species recognize and respond to snakes. Since the focus of our paper is on camouflage patterns and their relationship to prehistoric graphical expression, we will emphasize here the observations and experimental studies of snake-scale saliency. Humans are indeed capable detectors of snakes even under experimental conditions of blending camouflage (see Kawai and He, 2016).

In addition to their camouflaging properties on some snake species, snake-scale patterns can be visually conspicuous and aposematic, warding off snake predators and the inadvertent intrusions of benign species, such as foraging monkeys, due to their apparent mimicry of large, uncertain threats. This deterrent effect is apparent for the paired eyespots on ventral hood of the Indian cobra (*Naja naja*) that resemble two facing eyes. During experimental presentations to wild bonnet macaques (*Macaca radiata*) in southern India, an animated cobra model elicited startle or evasive flight response more than other animated snake models (Ramakrishnan et al., 2005). Another snake-scale example with aposematic properties is evident for the red- and yellow-ringed pattern of venomous coral snakes (*Micrurus* spp.). Presentations of elongated cylinders with these colored bands to hand-reared turquoise-browed motmots (*Eumomota superciliosa*) engendered alarm calling and avoidance by these birds (Smith, 1977).

Innate-recognition systems always require experience for their expression; that is, experientially driven neural activation in the appropriate situation is essential. For example, the unique neural pathways engendering snake recognition are latent in California ground squirrels (*Otospermophilus beecheyi*) living in snake rare or free habitats for many thousands of years. Yet, their relict neural circuits specialized for detecting and coping with snakes can be activated within seconds on first laboratory exposure to two of their historical snake predators: the northern Pacific rattlesnake (*Crotalus viridis oreganus*) and Pacific gopher snake (*Pituophis melanoleucus catenifer*; Coss, 1991, 1999). Such initial neural-circuit activation includes snake species discrimination involving auditory and olfactory modalities, with vision playing an important role. Moreover, the first day California ground squirrel pups use vision to navigate, they recognized a caged gopher snake and cautiously investigated a series of horizontal tick marks for measuring squirrel distance in the snake-presentation apparatus (Coss, 1991). This unexpected effect was fortuitous because the equally spaced tick marks superficially resembled the string of black spots on gopher snake scales. This cautious investigative response to repetitive tick marks also occurred during initial trials with a caged guinea pig (*Cavia porcellus*), indicating that snake odor did not prime this behavior. In the field, adult ground squirrels are capable of detecting tethered rattlesnakes within 10m (pers. obs.), but also shed rattlesnake skin within 2m since they will chew it and lick their fur as a possible deceptive defense against other predators (Clucas et al., 2008). As in ground squirrels, innate visual recognition of snakes is evident in laboratory born Chilean rodents (*Octodon degus*) that avoided a chamber with snake pictures (Watanabe et al., 2021).

Several species of captive-born New and Old World primates exhibit an innate ability to recognize snakes as dangerous. For example, Japanese macaques (*Macaca fuscata*) looked longer reliably at pictures of snakes than flowers (Shibasaki and Kawai, 2009) and captive-born pig-tailed macaques (*Macaca nemestrina*) differentiated a snake and lizard model by mild caution (Weiss et al., 2015). In large enclosures, captive-born rhesus macaques (*Macaca mulatta*) alarm called vigorously at a realistic model of an Indian python (*Python molurus*) placed outside their enclosures (Hollis-Brown, 2005). In later research at the same facility, rhesus macaques responded quietly to fully exposed and partially concealed snake models by standing quickly with bipedal postures to investigate these smaller snake models (Etting and Isbell, 2014). Alarm calling at pythons that eat monkeys characterized snake species discrimination by Indian macaques because wild bonnet macaques (*Macaca radiata*) only alarm called at an animate model of a python compared with other animate snake models that included venomous species (Ramakrishnan et al., 2005). van Schaik and Mitrasetia (1990) also observed alarm calling at pythons by wild long-tailed macaques (*Macaca fascicularis*).

While large snake size provides a simple cue for differentiating pythons from other snake species, differences in the snake-scale patterns among snakes might provide complementary information useful for distinguishing dangerous and harmless snakes. Wild white-faced capuchin monkeys (*Cebus capucinus*) in Costa Rica exhibited greater frequencies of alarm calling and level of vigilance toward two photographic models of a dangerous boa constrictor (*Boa constrictor*) and neotropical rattlesnake (*Cebus durissus*) compared with a white snake model control without any pattern (Meno et al., 2013). More subtle snake-scale discrimination was evident in further analyses of the acoustical structure of capuchin alarm calls documenting that these monkeys, notably the infants, distinguished the boa model, which exhibited a snake-scale pattern with sequential ovals, from a harmless scorpion eater (*Stenorrhina freminvillei*) that exhibited a uniform color without any pattern (Coss et al., 2019).

Less realistic repetitive scale patterns were still provocative to captive-born common marmosets (*Callithrix jacchus*) that alarm called while focusing their attention on serpentine and triangular clay models with crosshatching, crisscrossing stars, and striated lines (Wombolt, 2015; Wombolt and Caine, 2016); albeit, the serpentine models were reliably more provocative. A more explicit test of snake-scale detectability was conducted by Isbell and Etting (2017) on wild vervet monkeys (*Chlorocebus pygerythrus*) in central Kenya. These researchers exposed a narrow section of gopher snake skin to foraging monkeys and noted their enhanced wariness and memory of where they last saw the apparent snake. Follow-up research with captive titi monkeys (*Plecturocebus cupreus*) demonstrated a similar effect of sustained attention, comparing a small (2.5cm length) segment of gopher snake skin with a same-length feather (Lau et al., 2021).

**Figure 2:**
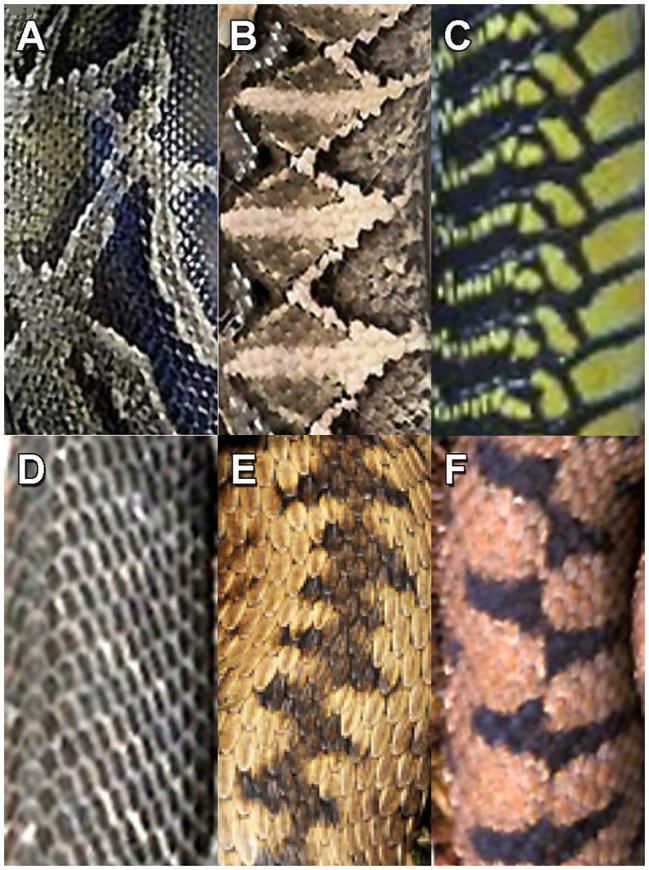
Patterns on the snake scales of dangerous snakes: **(A)** African rock python (*Python sebae*), **(B)** African Gaboon viper (*Bitis gabonica*), **(C)** African boomslang (*Dispholidus typus*), **(D)** African black mamba (*Dendroaspis polylepis*), **(E)** Common European viper (*Vipera berus*), and **(F)** European asp (*Vipera aspis*).

African rock pythons (*Python sebae*) along with venomous cobras and vipers have likely posed a threat to hominoid apes and hominins, including modern humans, since the early Miocene epoch (Headland and Greene, 2011). As a possible characterization of snake encounters by human ancestors, the type of response to the discovery of a python depends on the properties of surprise, proximity, and group behavior. Sudden python discovery by common chimpanzees (*Pan troglodytes*) can lead to alarm calling and flight to nearby trees; in contrast, chimpanzees initially alerted by alarm calling will investigate the snake cautiously with less provocative calling (*cf.* van Lawick-Goodall, 1968; Zamma, 2011). Informal interviews of herpetologists searching for snakes indicated that they were not frightened by intentional snake discovery. However, unexpected detection of nearby snakes still elicited the automaticity of immediate freezing or jumping back along with vivid recollections of these experiences (Penkunas and Coss, 2013a). Such memory vividness will be discussed further in the context of the aforementioned depictions of engraved crisscross and crosshatched patterns. In general, humans detect snakes relatively effectively during daytime, because the majority of snakebites occur at night when people step on them accidently (Nhachi and Kasilo, 1994). Examples of the dorsal scale patterns of venomous arboreal and terrestrial African snakes and terrestrial European vipers that require rapid detection by contemporary humans appear in Figure 2.

With regard to the developmental aspects of snake recognition by humans, it must be noted that overt fear of live snakes emerges slowly, typically after the second year (see Jones and Jones, 1928; Spindler, 1959) and coincidental with the onset of nighttime fear (Coss, 2021). Infants as young as 7months watching films of snakes compared with other animals did not show fearfulness; albeit, a suggestive precursor of fear is evident because a fearful human voice was associated with snakes (DeLoache and LoBue, 2009). Another possible precursor of snake fear is evident in 6–9-month-old infants that showed a slowing of heart rate, while watching a snake video compared with other images and a shorter latency to startle following a brief flash of a white video frame embedded in the video (Thrasher and LoBue, 2016). Another physiological measure, interpreted as a precursor of fear, is evident in 6-month-old infants that showed larger pupillary dilation, a sympathetic nervous system response, while viewing pictures of snakes and spiders compared with pictures of flowers and fish (Hoehl et al., 2017). These studies are augmented by evidence that infants and young children direct their gaze at images of snakes more quickly than at benign images and coiled snakes compared with various other coiled objects (e.g., LoBue and DeLoache, 2010, 2011).

### Saliency of Leopard Rosettes

Leopards (*Panthera pardus*) have likely posed a threat to human ancestors since the Middle Pliocene 3.8–3.6Ma (Treves and Palmqvist, 2007; Coss, 2021). Brain (1970, 1981) has described an incident in which an Early Pleistocene hominin was killed by a leopard interpreted from the spacing of lower-canine punctures in its skull. Leopard predation on early hominins roosting in trees and on the ground might have been high in forested areas with poor visibility. In such a setting, the likely predation rate on individual chimpanzees by leopards in one West African national park has been estimated to have occurred within 18years (Boesch, 1991, p. 235). If similar to extant African primates that react quickly to the sight of leopards (e.g., Busse, 1980; Isbell and Bidner, 2016), human ancestors needed to detect hunting leopards quickly for seeking refuge because leopards typically ambush from cover. In this context of stealthy leopard hunting, detection of crouching leopards partially occluded by vegetation would require rapid detection of salient visual features, such as two facing eyes, facial flecks, and exposed shoulder blades.

As described above, predator camouflage evolved initially to preclude detection by prey using optical blending with visually occluding vegetation and background illuminated by dappled light. Unlike snake-scale camouflage, however, leopard rosettes typically appear in semi-random arrays without the periodicities of snake scales. Nevertheless, leopard rosettes visible on partially occluded leopards appear to be salient predator recognition cues, partly aided by their yellowish background hue. In macaques, the neurons in early vision have been shown to be very sensitive to yellowish hues (see Yoshioka and Dow, 1996; Yoshioka et al., 1996). Such saliency in hue and pattern created the arms race for evolved perceptual enhancement of leopard detection by primate and ungulate prey (Ramakrishnan and Coss, 2000) and mitigation of such detection by stealthy daytime and nighttime hunting (*cf.* Zuberbühler and Jenny, 2002; Jenny and Zuberbühler, 2005; Isbell et al., 2018).

Experimental presentations of leopard models are informative of the role of rosettes in leopard recognition. For example, a brief 25m distance presentation of a realistic leopard model to wild bonnet macaques elicited flight-reaction times of 200–300ms in forest settings where leopards were present, as well as in a park-like university settings with monkeys that did not have previous experience with leopards (Coss and Ramakrishnan, 2000). Both sets of monkeys still responded, albeit slower and less vigorously, to the same model presented upside-down, whereas a model of a dark leopard morph without rosettes presented upside-down was much less provocative. Follow-up research on leopard-experienced forest monkeys (Coss et al., 2005) revealed that presentations of just the model leopard’s forequarters with facing head or just its hindquarters and tail, partially exposed from behind a bush, were still provocative, engendering alarm calling and flight to trees. No responses were made to presentations of the dark morph’s hindquarters and tail, again demonstrating the perceptual potency of leopard rosettes on a yellow coat (Coss et al., 2005, 2007).

Other studies are also informative. For example, leopard-naïve Guereza monkeys (*Colobus guereza*) roosting in trees in Uganda reacted promptly by alarm calling when they detected a person walking on all fours below wearing a yellow sheet with rosettes simulating a leopard (Schel and Zuberbühler, 2009). Leopard-naïve sooty mangabeys (*Cercocebus atys*), pigtail macaques, and rhesus macaques also exhibited vigorous alarm calling the first time they saw a leopard model (Davis et al., 2003). In a related study, captive-born rhesus macaques alarm called and climbed the walls of their tall enclosures when presented a model leopard (Hollis-Brown, 2005) resembling the model used by Coss and Ramakrishnan (2000).

### Experimental Questions and Hypothesis

While the aforementioned research examining infant attention provides suggestive evidence of innate snake recognition, nothing is known about how human infants respond to leopard rosettes. Pictures can be useful for evaluating the gaze behavior of infants, but the absence of overt fear in infants makes interpretation difficult (see critique of snake-presentation protocols by Tierney and Connolly, 2013). The saliency of images of snakes compared with lizards and lions compared with antelope had been studied previously in older American children and adults, and in comparative samples in southern India, allowing a comparison of urban children with limited wildlife experience to children living in forest areas, where pythons, venomous snakes, and leopards occur (see Penkunas and Coss, 2013a,b). Children in both cultures, as well as those living in urban and forests settings, were found to exhibit similarly shorter latencies in snake- and lion-image detection using a touch-screen display, suggesting that life history experiences did not account for snake and lion saliency. Since detection of snakes and leopards by non-human primates in the wild typically involves serendipitous discovery, an experimental approach examining self-initiated encounters with the patterns of these animals by infants might reveal inherent behavioral caution earlier in development than experimenter-manipulated presentations.

In the following experiment, we decided to examine the action patterns of infants engaging in exploratory investigation of toys in familiar daycare settings. Previous research studying infant mouthing behavior found that infants handled and mouthed gleaming stainless steel and glossy plastic plates more frequently than a plate with a dull surface finish (Coss et al., 2003). While glossy surface finishes are attractive to infants, engendering mouthing, objects with glossy python scales and leopard rosettes might engender mouthing hesitancy by displaying both a glossy-cue signifying water (Coss and Moore, 1990) and the visual cues of historical snake and felid predators. The natural context of infants exploring the floor on hands and knees and investigating and mouthing jars safely, without adult intervention, allowed us to evaluate any pattern-deterrent effects on infant mouthing.

We thus predicted that infants would be dissuaded from mouthing glossy jars with python and leopard patterns. Moreover, if these jars were indeed provocative, any hesitancy to handle them might be preceded by a tendency to look in the direction of nearby adults. Research on parental modeling has shown that the wariness of toddlers toward novel objects can be influenced by observing adult facial expressions (Gerull and Rapee, 2002). We also notice during preliminary observations that infants typically poked the jars with their forefingers before handling them. Exploratory poking has been observed in year-old infants (Blake et al., 1994) but, to our knowledge, has not been reported for younger infants. Hypothesizing that poking was a form of infant vigilance, we predicted that jars with python and leopard patterns would be poked more frequently than jars with geometric and plain patterns.

## Experiment 1

### Materials and Methods

#### Participants

Twenty-three infants and toddlers were observed at 3day-care facilities in the Sacramento Valley, California, spanning a period of 6months. Of these children, 14 (mean age=11.96months; range 7–15months) handled four patterned jars as experimental toys for five handling bouts in a repeated measures design derived from the plate-mouthing protocol reported by Coss et al. (2003). The Institutional Review Board of UC Davis approved the experimental protocol (Exemption 994,134), and informed consent was obtained from the parent of each participant.

#### Infant Interactions With Patterned Jars

**Figure 3:**
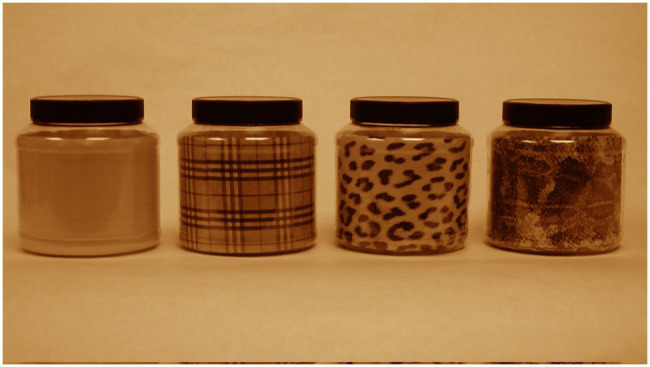
Jars displaying graphical patterns. From left to right: plain, Burberry plaid, leopard rosettes, and rock python scales.

Four glossy transparent plastic jars with black serrated lids were modified to present different patterns with the same background color pressed firmly against their side and bottom interior surfaces (Figure 3). These lightweight jars (136g) had the following dimensions: maximum dia.=14.5cm; maximum height with lid=15cm; height of interior pattern=11.5cm. Lid dimensions were: 11.3cm dia., height=2.2cm. Jar patterns were constructed of yellowish-orange paper providing the dominant background color (Munsell 7.5YR8/10). The following graphical patterns influencing infant handling were examined: (1) plain yellowish-orange color, (2) black and grayish-brown plaid (trademarked as Burberry plaid), (3) full-size black spots and rosettes of a leopard, and (4) full-size black and grayish-brown scale pattern of an African rock python. The plaid pattern was obtained by placing Burberry plaid cloth on a flatbed scanner. Similarly, the python-scale pattern was digitized by pressing the skin of a 3m rock python onto the scanner. The leopard texture was acquired from a close-up 35mm slide of a leopard’s flank. All digitized images were adjusted for contrast using Photoshop software.

#### Procedure

**Figure 4:**
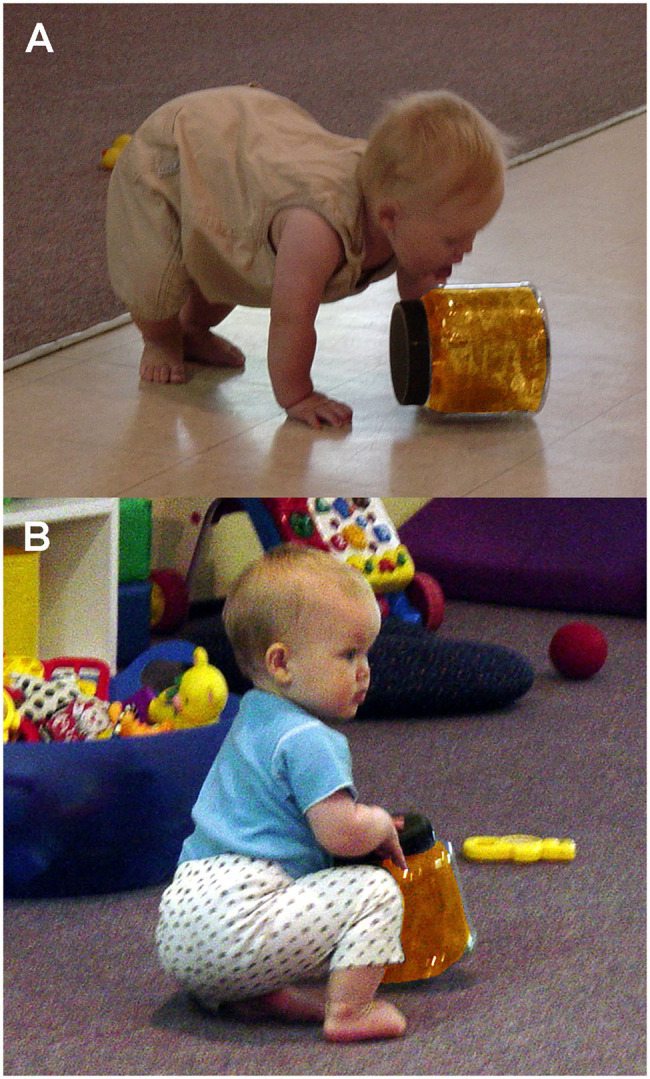
Examples of infant interactions with colored jars: **(A)** infant licking jar as a form of mouthing activity and **(B)** infant looking at adult while handing jar.

Tightly sealed jars were washed and dried prior to each observation period during children’s playtime. Each jar was placed near other toys in the play area in a balanced randomized order for a 5min observation period, thus yielding 20min sessions. Daycare attendants were instructed to treat the toys just like all other toys, and the observer was instructed to remain inconspicuous to the children. As in the earlier plate-mouthing study (Coss et al., 2003), mouthing (Figure 4A) was scored as a single event during each jar-handling bout. Also scored as single events just prior to a jar-handling bout were the behavioral measures of brief looking at a daycare attendant (Figure 4B) and probing the jar by poking it with an outstretched finger.

### Results

#### Qualitative Description

Again, analyses of infant behavior were restricted to those who handled the jars for five handling bouts. Because of their bulky shape, children held the jars with both hands and tended to mouth the rounded bottom edge. The average frequency of mouthing, which included licking the jars, was the highest for the Burberry plaid jar (20.6%) and the lowest for the plain jar (7.5%). The average frequency of looking at an adult prior to handling the jars was the highest for the python jar (29.8%) and the lowest for the plain jar (15.8%).

As mentioned above, poking was noticeable immediately in this experiment and was most apparent for the jars with the leopard and python textures. Poking included the careful prodding of the jars printed surface area typically with the outstretched index finger of the right hand. Poking usually remained brief, yet multiple children engaged in repeated poking of the same locus for several seconds. Each poke resulted in contact with the jar. When such behavior occurred, the jar was typically in an upright position. No poking occurred on the jar’s black serrated lid. The children who did poke the jars were typically either sitting next to the jar or facing the jar after crawling over to it. If the jar was on its side, poking would typically cause the jar to roll. Such jar movement would generally suspend the child’s interaction with the toy. Any poke was considered part of a handling bout. Hesitancy in further engagement with the jars as toys was especially evident when the two biologically textured jars were poked. Other than this suspension of playing with the jars, there was no observation of overt fearfulness during any interactions with the jars.

#### Quantitative Analysis

**Figure 5:**
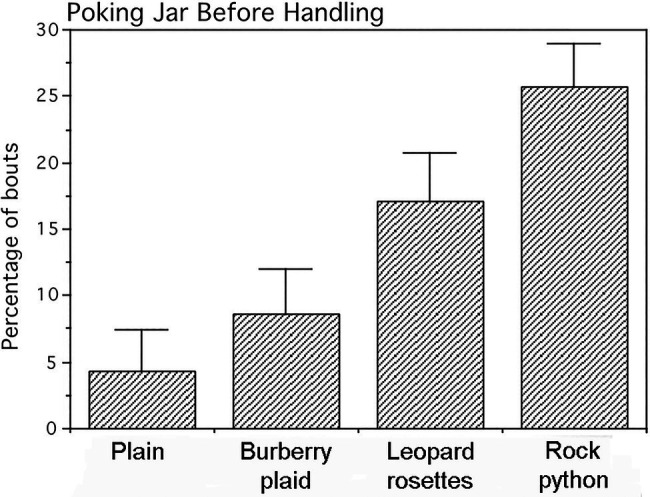
Percentage of jar-handling bouts in which infants poked the jars prior to handling them. Means and SEs are shown. The jars displaying leopard rosettes and the rock python-scale pattern were poked significantly more than the Burberry plaid and plain jars.

A single-factor repeated measures multivariate ANOVA with tests of simple effects as planned comparisons was applied to arcsine (angle) transformations of frequency data from the three behavioral measures. This transformed data for all jars exhibited normal distributions. The four jars did not differ significantly for either the frequencies of infant mouthing (*p*=0.25) or frequencies of looking at adults prior to handling the jars (*p*=0.34). In contrast, the jars engendered statistically significant differences in poking frequency (Pillai–Bartlett trace=0.815, multivariate *F*_(3,11)_=16.135, *p*<0.00025). Planned comparisons revealed that the frequency of poking the plain and plaid jars (Figure 5) did not differ appreciably (*F*_(1,13)_=1.449, *p*=0.250). However, the leopard and python jars were poked at significantly higher frequencies with large effects sizes than either the plain or plaid jars [plain vs. leopard (*F*_(1,13)_=12.226, *p*<0.005), Cohen’s *d*=1.9; plain vs. python (*F*_(1,13)_=53.890, *p*<0.0001), Cohen’s *d*=4.1; plaid vs. leopard (*F*_(1,13)_=6.198, *p*<0.05), Cohen’s *d*=1.4; and plaid vs. python (*F*_(1,13)_=15.700, *p*<0.0025), Cohen’s *d*=2.2]. Although, the python jar was poked at a higher frequency than the leopard jar was poked, this difference only approached significance (*F*_(1,13)_=3.850, *p*=0.072; Cohen’s *d*=1.1).

Our next experiment employs an animate presentation of essentially the same patterns to younger infants with the expectation that snake scales and leopard rosettes will attract visual attention more than the Burberry plaid and plain patterns. To further our understanding of how gaze behavior influences motor activity, we also examine infant grasping behavior to evaluate any hesitancy to touch these patterns.

## Experiment 2

### Materials and Methods

#### Participants

Fifteen infants, spanning 5months of age, were studied for their gaze and reaching behavior. Infants were selected from a larger pool for their ability to self-sit and reach since this behavior emerges between 5 and 6months of age (see Rochat and Goubet, 1995). They were recruited primarily through mailings, supplemented by advertising flyers distributed to daycare centers, pediatric offices, and other community locations. Parents participated in the study by supporting their infants on their laps in front of a model presentation table. Parents received an infant T-shirt for participating. The Institutional Review Board of UC Davis approved the experimental protocol, and informed consent was obtained from the parent of each participant.

#### Presentation of Patterned Cylinders

**Figure 6:**
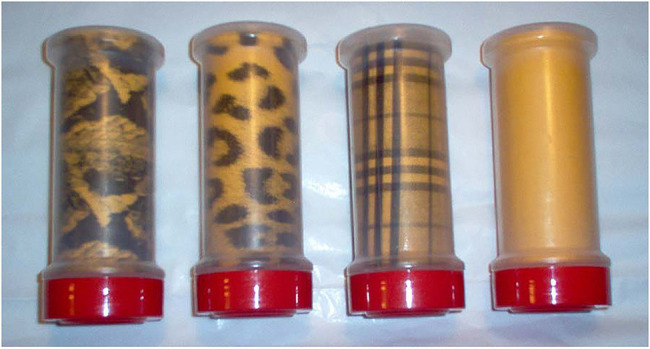
Cylinders displaying graphical patterns. From left to right: Gaboon viper snake-scale pattern, leopard rosettes, Burberry plaid, and plain.

Four transparent plastic cylinders were presented to infants. These cylinders were 13.5cm long, 4.7cm in diameter, and weighed 119g. Cylinder bases were weighted so they stood upright, and the 5.5cm diameter rims at the tops and bases facilitated infant gripping. Each cylinder contained coiled paper pressed against the interior walls displaying the same dark-orange color (Munsell 7.5YR8/10) used in Study 1. Each cylinder exhibited a different pattern to examine. A snake-scale pattern was photographed from a live Gaboon viper (*Bitis gabonica*) as an alternative to using a larger rock python snake pattern. However, the leopard rosette pattern was adapted from Study 1 as were the Burberry plaid and plain patterns (Figure 6). Based on Photoshop pixel scanning, cylinder-luminance histograms from the highest to lowest mean values were as follows: plain cylinder=188, SD=16, leopard rosettes=112, SD=67, Burberry plaid=102, SD=63, and Gaboon viper=93, SD=56.

A 76×76cm table was used with the researcher sitting on one side and the parent with the infant on her lap centered on the other side. One side of the table had an 18-cm-high×76-cm-long transparent acrylic plastic wall to keep the infant from throwing or bumping the cylinders off the table.

**Figure 7:**
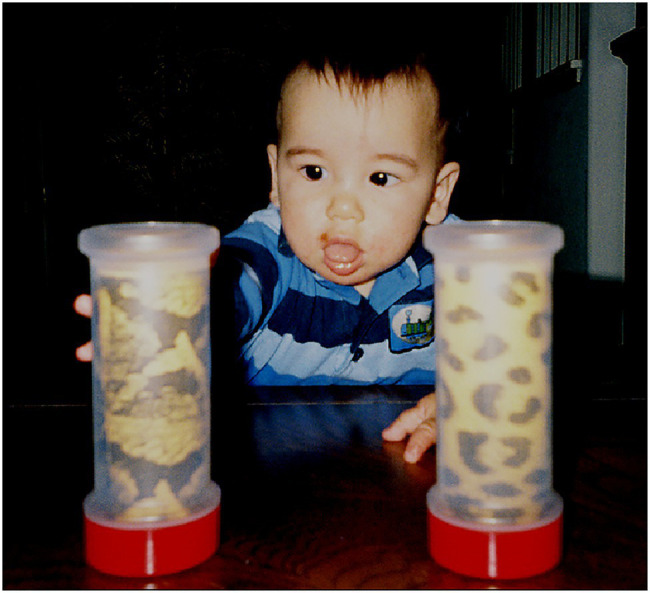
Infant reaching for a cylinder displaying the Gaboon viper snake-scale pattern after both cylinders were positioned by the experimenter.

To begin the experiment, the infant was centered on her/his parent’s lap, facing the experimenter and the video camera, with all four cylinders out of view. Each infant was presented two cylinders simultaneously in a paired comparison procedure. All paired comparisons were reversed so infants observed the same cylinder on their left and right sides, thus yielding six presentation trials per cylinder and 24 paired comparisons overall. Cylinder presentations consisted of the experimenter placing two cylinders on the table, visually aligned with the infant’s shoulder. The experimenter then pushed them simultaneously by their bottom red caps in a parallel trajectory toward the infant at a slow even pace until they were within the infant’s grasping reach as shown in a post-experiment example (Figure 7). The average cylinder pushing time was 5.989s calculated from the video recordings. At this point, the infant grasped a cylinder and typically proceeded to put the top portion of the cylinder in her/his mouth (all cylinders were sanitized after each infant’s use).

#### Gaze Behavior and Grasping Quantification

A tripod-mounted video camera (Panasonic model PV-DV601D with a 20× zoom lens) was positioned behind the researcher to video record infant direction of gaze and cylinder-grasping behavior. Recordings were copied using a Panasonic FA-400 time base corrector, with each video field labeled numerically by a FOR.A VTG-22 video field number generator. The duration (milliseconds) that each infant looked at the cylinders was converted to a percentage of time that the cylinders were moving toward this infant to account for any variation in pushing speed and starting distance. This time frame was measured using video slow-motion and field-by-field playbacks on a Panasonic video-editing deck with a 21in monitor to measure left and right saccadic shifts of infant gaze in 16.67ms increments. This procedure circumvented the issue of computer delays from eye-tracker hardware and software (see Aslin, 2012).

### Results

**Figure 8:**
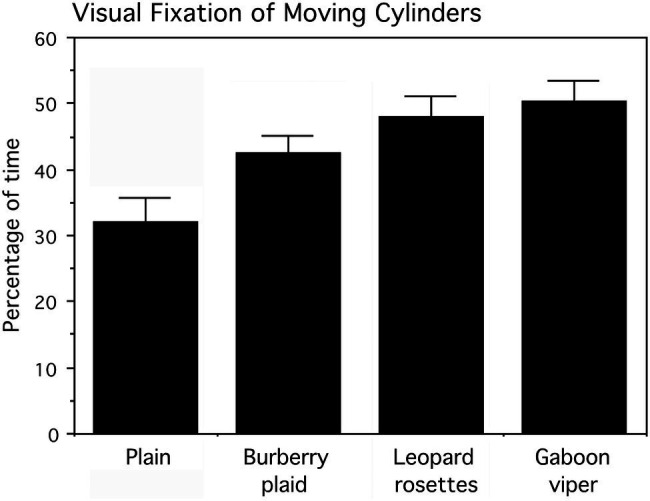
Percentage of time, averaged for six presentation trials that infants looked at the cylinders as they were pushed toward them by the experimenter. Means and SEs are shown. The cylinders displaying leopard rosettes and the Gaboon viper snake-scale pattern were looked at significantly longer than the Burberry plaid and plain cylinders.

Infants reached for the cylinders predominantly with their right hands (57.99% of the time; Wald *χ*^2^_(1)_=4.296, *p*=0.039). The frequencies for cylinder grasping are, respectively, left hand=71 times and right hand=98 times of 180 trials. There was no evidence that infants avoided reaching for the cylinders with biological patterns. The percentage of time each infant visually fixated the cylinder as it was pushed was subjected to an arcsine transformation for statistical analyses. Actual percent values (means and SEs) for cylinder visual fixation are presented in Figure 8. The cylinder grasped was also noted for logistic regression analysis.

A two-factor (four cylinders and six trials averaged for the infant’s left and right sides) repeated measures ANOVA examined the percentage of time infants looked at the cylinders, followed by tests of simple effects comparing all cylinders. The main effect for cylinders, averaged for trials, was statistically significant (*F*_(3,42)_=5.454, *p*=0.003), whereas the main effect for trials (*F*_(5,70)_=0.528, *p*>0.5) and the interaction of cylinders and trials (*F*_(15,210)_=0.901, *p*>0.5) were not statistically significant. As apparent in Figure 7, the biological patterns were fixated visually more than the plaid and plain cylinders as they moved toward the infants. The mean percentage of visual fixation time was the highest for the Gaboon viper cylinder, followed closely by the leopard rosette cylinder. Averaged for trials, a planned comparison of these biological patterns indicated that they did not differ appreciably (*F*_(1,14)_=0.447, *p*>0.5). However, both the Gaboon viper cylinder and leopard rosette cylinder were looked at for significantly longer percentages of time than the non-biological Burberry plaid cylinder (respectively, F_(1,14)_=4.861, *p*=0.045, and 4.898, *p*=0.044). The standardized effect sizes for these mean differences are large (Cohen’s *d*=1.6 for the Gaboon viper–Burberry plaid comparison and *d*=1.2 for the leopard rosette–Burberry plaid comparison). In addition, the plain cylinder was looked at significantly less than the Gaboon viper cylinder (F_(1,14)_=8.989, *p*=0.010; *d*=1.6) and the leopard rosette cylinder (F_(1,14)_=6.025, *p*=0.028; *d*=1.3), but not the Burberry plaid cylinder (F_(1,14)_=2.985, *p*=0.106).

### The Effect of Gaze Duration on Cylinder Choice

Because, we did not measure visually guided reaching due to our focus on cylinder differentiation while cylinders were in motion, our evaluation of cylinder saliency on cylinder choice is restricted to the effect of gaze duration prior to reaching. As such, logistic regression was used to assess whether the percentage of time looking at each cylinder, while it was pushed toward the infant influenced whether that cylinder was grasped. Since the cylinders were presented on the infant’s left and right sides, each cylinder in the paired comparison was examined separately by logistic regression to circumvent any side bias. The arcsine percentage of visual fixation time, while each cylinder was in motion was the predictor variable, and the presence or absence of cylinder grasping on the same side was the dependent (response) variable (Table 1). Seventeen of 24 logistic regressions indicated statistically significant relationships between the durations of looking at a cylinder prior to grasping it.

**Table 1:** Logistic regressions of moving cylinders in paired comparisons grasped by infants on their right or left sides and whether their percentages of time looking at these cylinders predicted whether they were grasped.

Cylinder looked at and grasped	*χ* ^2^	Odds ratio	*p*
1a. Gaboon viper (right) selected over Leopard (left)	8.333	1.115	0.00389
1b. Leopard (left) selected over Gaboon Viper (right)	4.897	1.076	0.02691
2a. Gaboon viper (left) selected over Leopard (right)	2.714	1.060	0.09948
2b. Leopard (right) selected over Gaboon Viper (left)	1.214	1.036	0.27052
3a. Gaboon viper (right) selected over Burberry Plaid (left)	9.089	1.134	0.00257
3b. Burberry Plaid (left) selected over Gaboon Viper (right)	10.931	1.232	0.00094
4a. Gaboon viper (left) selected over Burberry Plaid (right)	0.052	1.005	0.81895
4b. Burberry Plaid (right) selected over Gaboon Viper (left)	0.010	1.002	0.92205
5a. Gaboon viper (right) selected over plain (left)	1.010	1.044	0.314853
5b. Plain (left) selected over Gaboon Viper (right)	5.252	1.143	0.021933
6a. Gaboon viper (left) selected over plain (right)	13.118	1.179	0.000293
6b. Plain (right) selected over Gaboon Viper (left)	8.326	1.070	0.003911
7a. Leopard (right) selected over Burberry Plaid (left)	8.595	1.172	0.003373
7b. Burberry Plaid (left) selected over Leopard (right)	3.270	1.048	0.070555
8a. Leopard (left) selected over Burberry Plaid (right)	10.159	1.120	0.001438
8b. Burberry Plaid (right) selected over Leopard (left)	20.054	2.353	0.000008
9a. Leopard (right) selected over plain (left)	4.593	1.080	0.032109
9b. Plain (left) selected over Leopard (right)	0.061	1.009	0.804780
10a. Leopard (left) selected over plain (right)	16.793	3.318	0.000042
10b. Plain (right) selected over Leopard (left)	16.197	2.448	0.000057
11a. Burberry Plaid (right) selected over plain (left)	9.003	1.157	0.002697
11b. Plain (left) selected over Burberry Plaid (right)	14.545	1.781	0.000137
12a. Burberry Plaid (left) selected over plain (right)	18.139	2.484	0.000021
12b. Plain (right) selected over Burberry Plaid (left)	7.665	1.078	0.005633

The seven looking and grasping relationships with non-significant values all involved cylinders with snake scales or leopard rosettes on the opposite sides that competed for attention prior to the infant’s decision to reach for one of the cylinders. The largest mean values for gaze were when the three patterned cylinders were presented on the right side, favoring grasping by the right hand. However in Table 1: 4a, the Gaboon viper cylinder presented on the left side was selected over the Burberry plaid cylinder on the right side even though the Burberry plaid cylinder was grasped by 66.67% of the infants. Conversely in Table 1: 4b, when the Gaboon viper cylinder was presented on the infant’s right side and the Burberry plaid cylinder was presented on the infant’s left side, the Gaboon viper cylinder attracted the infant’s attention prior to reaching with the highest acrsine mean value (59.54%) among all paired comparisons and the second-highest percentage (66.67%) of overall cylinder grasping by the infants. This particular paired comparison suggests that infants were not cautious or inhibited in grasping the Gaboon viper cylinder. Visual competition prior to reaching appeared to occur in the paired comparison when the Gaboon viper cylinder was presented on the infant’s left side, and the leopard rosette cylinder was presented on the infant’s right side or vice versa (Table 1: 2a,b). Logistic regression of each cylinder in this set of paired comparisons indicated that visual fixation prior to reaching did not predict reliably which cylinder was grasped.

Finally, it must be noted that visual fixation of the Burberry plaid and plain cylinders engendered very strong predictive relationships with cylinder grasping, especially when the Burberry plaid was presented on the left side and the plain cylinder was presented on the right side, leading to 60% of infants grasping the Burberry plaid cylinder on their left side typically with their right hand (Table 1: 12b). A similar reaching over occurred when the Gaboon viper cylinder and leopard rosette cylinder were presented on the left side and the plain cylinder was on the right side (Table 1: 6a and 10a). Together, it is apparent that all three patterned cylinders presented on the left side caught the infant’s attention in ways prior to reaching that engendered grasping them on the infant’s less favored side.

## Discussion

Counter to our predictions, none of the jars in Experiment 1 were distinguished by the frequency of mouthing the glossy plastic or looking first at adults prior to handling the jars. The jars *did* differ reliably in the frequency of poking them with an extended index finger. Again, poking as exploratory behavior has been reported for some 12month olds at an object with protruding features (see Blake et al., 1994, p. 197). If interpreted correctly as a probing action, the jars with python and leopard patterns created the most uncertainty for investigative infants, especially if the jars rolled after being poked. Our only anecdotal observation of fear of any of our jars was that of an 18-month-old non-participant girl who shouted “NO! NO!,” while pointing to the jar with leopard rosettes after she spotted it on the floor.

The absence of fear of any of the jars at 7–15months of age warrants further discussion. As mentioned above, Spindler (1959) reported that young children exhibited fear of a live snake as early as 2years of age, although Jones and Jones (1928) used the term “guarded” as their description of hesitant investigation of live snakes as early as 26months of age. Taken together, it appears that this developmental delay in the overt expression of snake fear has anticipatory properties characterized by greater attention toward snake images and subtle physiological measures of arousal (Thrasher and LoBue, 2016; Hoehl et al., 2017). However, it must be noted that this developmental delay is not apparent when infants see unfamiliar humans because fear of strangers typically begins about 8months of age (e.g., Schaffer, 1966; Scarr and Salapatek, 1970).

A paired comparison procedure was used in Experiment 2 to determine whether graspable cylinders displaying the scales of a Gaboon viper, rosettes from a leopard coat, a commercial Burberry plaid, and a plain surface differed appreciably in attracting the attention of 5-month-old infants whose visuomotor coordination of reaching was undergoing developmental refinement (see von Hofsten, 1984; Rohlfing et al., 2013). Because formerly camouflaging skin and coat pattern of animals dangerous to human ancestors might act as recognition cues, cylinders depicting snakes scales and leopard rosettes were predicted to capture attention longer as they were pushed toward each infant. None of the infants hesitated in grasping any of the cylinders, unlike the poking action by older infants observed in Experiment 1. Nevertheless, the results of both experiments showed the same relational trend in their dependent measures, with infants responding the most to snake scales, followed by the leopard rosettes, Burberry plaid, and plain patterns. Such consistency in this progression of responsiveness suggests that the two ecologically relevant patterns might have inherent significance to humans prior to the developmental manifestation of overt fear.

In other mammalian species, rapid visual information processing for survival purposes entails the superior colliculus (SC), an ancient subcortical structure enlarged in diurnal rodents (e.g., Woolsey et al., 1971) that might play an important role in visual predator recognition, including snakes (Sewards and Sewards, 2002). In contrast, the SC in arboreal primates is small relative to neocortical volume, seemingly retaining much of its earlier size during the early evolutionary shift in neocortical enlargement (e.g., Bons et al., 1998). For example, in macaque monkeys, the SC functions to coordinate gaze behavior with arm movement during reaching (see Stuphorn et al., 2000). Despite SC coordination of gaze and reaching, it is unlikely that the SC performed the pattern recognition tasks that directed infant attention toward the cylinders with snake scales and leopard rosettes even though human SC neurons can be activated by a flickering checkerboard pattern (Schneider and Kastner, 2005).

Our argument about restricted SC pattern recognition capability is based on neurophysiological findings of macaques. First, the percentage of retinal ganglion cells projecting to the SC in humans would not likely exceed the approximately 10% of all retinal projections found in macaques (Perry and Cowey, 1984). Even with sparse foveal input, this level of retinotectal connectivity would yield coarse pattern perception by the SC with a low spatial frequency that might not be sufficient to resolve snake scales as recognition cues from partially occluded snakes and leopard rosettes on partially exposed leopards at distances known to elicit alarm calling. Nevertheless, the course visual resolution of the human SC is sufficient to engender amygdala activation during the blurry perception of fearful faces *via* the subcortical colliculo–pulvino–amygdala pathway (Rafal et al., 2015; Burra et al., 2019).

Secondly, besides the limited retinotectal input in higher primates, there is another source of input to the superficial layers of the SC capable of processing complex shapes at higher resolution – the visual and middle temporal cortices (*cf.* Cerkevich et al., 2014; White et al., 2017). This ability is complemented by an important neural structure involved in visual attention, the pulvinar, a thalamic nucleus synchronizing the neural activity of other brain areas (Saalmann et al., 2012). Evidence for the argument for involvement of the SC in snake recognition is that neural recordings from the macaque pulvinar show differential activity to images of snakes in striking postures compared with non-striking postures (Le et al., 2014). However, pulvinar neural responses to snakes could well reflect visual information from the dense projections from medial and inferotemporal (IT) cortices, characterizing a brain region homologous with the fusiform face area in humans, where cortical face processing occurs along with unrelated shapes in macaque IT (Benevento and Miller, 1981; Tanaka, 2003). Therefore, the pattern recognition capabilities by the primate SC are ambiguous, beyond that of rough face perception with two facing eyes (Nguyen et al., 2014) that is much more evolutionarily refined in the human fusiform face area and occipital face area (*cf.* Arcurio et al., 2012; Cecchini et al., 2013; Parkington and Itier, 2018). Nevertheless, the SC does play an important role in behavioral coping with dangerous animals due to its deep-layer projections to the midbrain periaqueductal gray (PAG) that initiates rapid freezing and directed flight behavior (Mantyh, 1982; Dean et al., 1989; Wei et al., 2015).

Detail neurophysiological study of snake perception in visual and temporal cortices has not been conducted to our knowledge, but there are indirect findings suggestive of the ability to process snake- and leopard-like features. For example, Kastner et al. (2000) report a gradual increase in neural responsiveness to presentations of texture-defined contours using fMRI in humans as one progresses from early vision to higher-order visual areas. More relevant to our discussion of snake-scale perception, Okusa et al. (2000) used magnetoencephalography with repeated pattern presentations to humans and showed that diamond and cross-patterns activated neurons the ventral extrastriate cortex neurons that include the fusiform face area with a faster latency and greater amplitude than alternating black or white clusters of dots randomly changing in configuration. More recent human research measuring event-related potentials (ERPs) from scalp electrodes has shown similar effects by presenting photographs of snakes with prominent triangular-like scale patterns that evoked more sustained attention characterized by late (250–350ms) negative potentials of the right-frontal hemisphere compared with snakes without patterns (Grassini et al., 2019, p. 67).

Often used as comparative controls, checkerboard patterns exhibit the periodicity of snake scales. ERPs of epilepsy patients recorded from surfaces of the ventral visual cortex showed that a checkerboard pattern elicited strong 100ms ERPs (Allison et al., 1999, p. 417). Based on this 100ms ERP finding in human visual areas, known to project to the SC in macaques (Cerkevich et al., 2014) that activates PAG defensive behavior, it is reasonable to argue that the 233–267ms reaction times of bonnet macaque freezing, standing, or jumping back after discovering snakes (Ramakrishnan et al., 2005) is consistent with the activation of integrative neocortical and subcortical neural pathways. Also suggestive of longer automatic attention to snakes, the early posterior negativity of ERPs from scalp electrodes is more sustained after viewing images of snakes than after viewing images of lizards and angry and neutral faces (Langeslag and van Strien, 2018).

While there is an emerging neurophysiological and behavioral literature on snake detection and recognition, in a variety of species including humans, there is no experimental evidence on how adult humans respond behaviorally to sudden discovery of a leopard in a more natural context. There are published descriptions of human–leopard interactions (e.g., Corbett, 1947) and numerous unpublished anecdotes from researchers in the field and from tourists on game drives. Research on preschool children conducted under adult supervision has shown that the sudden appearance of a realistic model leopard is more provocative than a model deer, engendering hesitation to seek refuge away from adults in a playground setting (Coss and Penkunas, 2016). While not specific to leopards, neurophysiological research has been approximated, in which image saliency was inferred from the maximum responses of a macaque neuron in anterior inferotemporal cortex to a drawing of a striped cat and horizontal and vertical gratings and another neuron’s responses to a cat’s striped hind leg and a stripe-like vertical grating (Tanaka et al., 1991; Tanaka, 2003, p. 91). Moreover, Tanaka et al. (1991, p. 177) reported other texture-sensitive neurons highly responsive to an orderly arrangement of spots.

## Study Limitations

The protocol for Experiment 1 required observers to record three types of focal-infant behavior as single events for each jar-handing bouts in free play situations. The stochastic aspects of infants playing with all the patterned jars for five jar-handing bouts yielded only 14 of 23 infants for repeated measures statistical analyses that reflect marginal convergences toward the true population means for all the jars. Although rigorous in its simplicity, focal-infant sampling has the limitation of missed observations if the observation intervals are too long, making it difficult for the observer to maintain her attention (see Altmann, 1974; Vonk, 2018). As an alternative, video recordings would have allowed detailed examination of infant playtime behavior in a familiar daycare setting, but this was not permitted due to a lack of unanimous caregiver approval at each daycare facility.

Video recordings of infant gaze and cylinder grasping were conducted in Experiment 2 in a laboratory setting. As with Experiment 1, the primary constraint on the interpretation of findings is the small sample size coupled with the limitation of 24 paired comparison cylinder presentations to prevent infant fatigue (see Kidd et al., 2012). Subsequent research should focus on comparing the biologically provocative patterns with those exhibiting similar complexity and fewer geometric features. However, the issue of image complexity is difficult to assess let alone quantify (*cf.* McCall, 1974; Chipman and Mendelson, 1975; Redies et al., 2017).

The aforementioned heterospecific evidence of snake and leopard recognition suggests that these perceptual systems evolved for distinguishing ecologically important static contours and patterns from similarly complex backgrounds as apparent when snakes and leopards freeze deliberately to avoid detection. Our first experiment combined both static and dynamic motion as when participants were initially attracted to the jars and then poked them. Our second experiment involved the visual tracking of moving targets against a moving background (presenter forearms) which does not evaluate the interaction of static and dynamic images. Future research could evaluate the interaction of moving contours and patterns presented behind different slit-like static backgrounds as if “painting” the figure across the retina (*cf.* Parks, 1965; Haber and Nathanson, 1968).

## Summary and Conclusion

As apparent from our literature review of non-human species, the ability to detect and recognize snakes quickly and engage in appropriate defensive behavior are perceptual and behavioral traits shaped by long periods of natural selection (Isbell, 2009). Similarly, rapid detection and recognition of leopards as dangerous predators coupled with evasive behavior have been under strong natural selection, but for a shorter evolutionary time span. The aforementioned studies of rodent and primate behavioral development, coupled with studies of captive-born prey species or species studied in predator-rare or free habitats, provide unambiguous evidence that snake and leopard recognition has innate perceptual properties. Because these recognition systems are latent, awaiting activation under appropriate experiential conditions, the timing of their expression can be limited by developmental constraints. For example, infant bonnet macaques still attached to their mothers have been observed to monitor snakes, then to release their grip, and approach them fearlessly, leading to the mother’s intervention by quickly grabbing the infant (Coss, 2003; Ramakrishnan et al., 2005). In contrast, older juveniles exhibit adult-like freezing, standing, or jumping back when a nearby snake is detected. Infant white-faced capuchins will alarm call at other animals, including snakes, beginning about 11months of age (Meno, 2012), so this delay in snake-directed fear is not unique in non-human primates.

### Implications of Image Saliency and Visual Imagery for Graphical Expression

The idea of linking artistic expression to evolved perceptual abilities is not new (e.g., Schlosser, 1952; Coss, 1968, 2003; Eibl-Eibesfeldt, 1989; Aikens, 1998a,b; Sütterlin, 2003; Watson, 2011; Orians, 2014; Mühlenbeck and Jacobsen, 2020). Outside of research on face perception in newborn infants (Goren et al., 1975; Johnson, 2005; Johnson et al., 2015), it is difficult to demonstrate unambiguous innate perception in humans unlike that of captive-born species or species living in predator-rare or predator-free habitats. Our experiments on young infants using biological textures that are evocative in ground squirrels and non-human primates do provide evidence that their saliency might influence the expression of crosshatching, zigzags, and dots that are common graphical designs in prehistoric art. Actual demonstration of this relationship requires further research on how visual imagery is translated into artistic production. Our following discussion about how these specific biological patterns influenced prehistoric graphical engravers is a speculative integration of experimental findings of memories and emotions.

Engraving hard surfaces involves a much slower process of hand–eye coordination than targeted drawing movements, and it requires a sustained concentration of where the gouging or scraping tool is directed on the surface. The vividness of mental images in working memory would play an essential role in exteriorizing these images onto worked pieces. Could the saliency of snake scales and rosettes be formative in a manner that enhances mental imagery of these patterns? One such process of augmenting the vividness of these mental images would be events remembered explicitly in autobiographical memory.

Flashbulb memories are a vivid form of autobiographical memory typically occurring when individuals experience surprising consequences coupled with intense emotions (Brown and Kulik, 1977; Rubin and Kozin, 1984). Memory rehearsal is not necessary for the clarity of flashbulb memories, and Yonelinas and Ritchey (2015) argue that the amygdala mediates “emotional binding” of observed events or images leading to a slow decay of event or image recollection. Evidence of SC and pulvinar involvement in snake perception would likely include the contribution of the amygdala in memory enhancement. Such emotional consequences leading to vivid mental imagery would have occurred mostly during unexpected encounters with snakes and dangerous felids throughout human evolution. From a life history perspective, it is probable that Upper Paleolithic and Middle Stone Age artists experienced daunting encounters with snakes and large-bodied felids, including leopards, and European cave lions (*Panthera spelaea*) that exhibited rosettes (Coss, 2020). From the neurophysiological perspective, the sudden detection of a nearby snake would involve rapid assessment of visual detail by extrastriate cortex and blurry image evaluation by the SC that activates rapid defensive behavior by the midbrain PAG (see Almeida et al., 2015; Konoike et al., 2020). More detailed appraisal of the snake, such as its configuration and distance, would follow quickly if the perceiver remained physically frozen or had jumped back.

Vivid mental images of snakes are associated with more intense snake phobia. When requested to explain what would happen if a snake appeared suddenly, individuals with self-reported fear of snakes imagined “vivid and horrifying” encounters (Hunt et al., 2006). Moreover, in non-phobic individuals, pictures of the common European viper with a zigzagging scale pattern and the Gaboon viper with a crisscross scale pattern (see examples in Figures 2B,E) were rated as more beautiful than pictures of non-dangerous snakes (Landová et al., 2018). Anecdotal commentaries of dangerous animals, such as leopards, as beautiful are not unusual. Consistent with the idea that beauty and danger are linked, Coss (2003, p. 83) proposed that the cognitive relationship between beauty and level of dangerousness might reflect a motivational system to maintain awareness of where dangerous animals or thorny plants might be encountered in the landscape (for a related perspective, see Landová et al., 2012). Beauty can also be associated with positive awe that enhances parasympathetic arousal and that is distinct from threat-based awe that increases sympathetic arousal, preparing the organism for action (Gordon et al., 2017). It is interesting to note in the context of threat-based awe that Wik et al. (1993) reported higher cerebral blood flow in the temporal lobes, but lower blood flow in the orbitofrontal cortex and prefrontal cortex, while snake-phobic individuals viewed a video of snakes. Due to the importance of snakes to phobic individuals, such an underactivation of orbitofrontal and prefrontal cortices during snake perception might enhance attentional control for appropriate action by reducing cognitive interference (see Northern, 2010).

Organized pattern engraving mediated by vivid mental images, possibly derived from flashbulb-like autobiographical memories of life history experiences, reflects one of the earliest features of creative thought during hominin evolution. Beginning with late *H. erectus* in Asia, engravings by prehistoric artists exhibit organized crosshatching that roughly resembles the periodicities of snake-scale patterns, while the later decorative spots by early modern humans possibly characterize the saliency of leopard rosettes. The findings of our experiments on infants provide an empirical context for future work on this topic.

## Data Availability Statement

The raw data supporting the conclusions of this article will be made available by the authors, without undue reservation.

## Ethics Statement

The studies involving human participants were reviewed and approved by The Institutional Review Board of UC Davis. Written informed consent to participate in this study was provided by the participants’ legal guardian/next of kin. Written informed consent was obtained from the minor(s)’ legal guardian/next of kin for the publication of any potentially identifiable images or data included in this article.

## Author Contributions

RC conceived and designed the experiments, created the graphical patterns, conducted statistical analyses, and wrote the paper with critique and further input from EC. EC recruited the infants for Experiment 2 and conducted part of the experiment. All authors contributed to the article and approved the submitted version.

## Funding

This research was supported by Faculty Research Grant D922 and an undergraduate teaching fund to RC from the University of California, Davis.

## Conflict of Interest

The authors declare that the research was conducted in the absence of any commercial or financial relationships that could be construed as a potential conflict of interest.

## Publisher’s Note

All claims expressed in this article are solely those of the authors and do not necessarily represent those of their affiliated organizations, or those of the publisher, the editors and the reviewers. Any product that may be evaluated in this article, or claim that may be made by its manufacturer, is not guaranteed or endorsed by the publisher.
